# Graphene Oxide Concentration Effect on the Optoelectronic Properties of ZnO/GO Nanocomposites

**DOI:** 10.3390/nano10081532

**Published:** 2020-08-05

**Authors:** Issam Boukhoubza, Mohammed Khenfouch, Mohamed Achehboune, Liviu Leontie, Aurelian Catalin Galca, Monica Enculescu, Aurelian Carlescu, Mohammed Guerboub, Bakang Moses Mothudi, Anouar Jorio, Izeddine Zorkani

**Affiliations:** 1 Group of Nanomaterials and Renewable Energies, Laboratory of Solid State Physics, Faculty of Sciences Dhar el Mahraz, Sidi Mohammed Ben Abdellah University, P.O. Box 1796, Atlas Fez 30 000, Morocco; boukhoubza.issam00@gmail.com (I.B.); achehboune.mohamed01@gmail.com (M.A.); mohammedguerboub@gmail.com (M.G.); a_jorio@hotmail.com (A.J.); izorkani@hotmail.com (I.Z.); 2 Africa Graphene Center, Department of Physics, College of Science, Engineering and Technology, Science Campus, University of South Africa, Cnr Christiaan de Wet & Pioneer Avenue, Florida 1709, Johannesburg, South Africa; khenfouch@yahoo.fr; 3 Faculty of Physics, Alexandru Ioan Cuza University of Iasi, Bulevardul Carol I, nr. 11, 700506 Iasi, Romania; lleontie@gmail.com; 4 Laboratory of Multifunctional Materials and Structures, National Institute of Materials Physics, Atomistilor 405A, 077125 Magurele, Romania; ac_galca@infim.ro; 5 Integrated Center for Studies in Environmental Science for North-East Region, Alexandru Ioan Cuza University of Iasi, Bulevardul Carol I, nr. 11, 700506 Iasi, Romania; aurelian.carlescu@uaic.ro; 6 Department of Physics, University of South Africa, Private Bag X90, Florida 1710, South Africa; mothubm@unisa.ac.za

**Keywords:** ZnO NRs/GO nanocomposites, hydrothermal method, CIE diagram, optoelectronic properties

## Abstract

In this work, the effects of graphene oxide (GO) concentrations (1.5 wt.%, 2.5 wt.%, and 5 wt.%) on the structural, morphological, optical, and luminescence properties of zinc oxide nanorods (ZnO NRs)/GO nanocomposites, synthesized by a facile hydrothermal process, were investigated. X-ray diffraction (XRD) patterns of NRs revealed the hexagonal wurtzite structure for all composites with an average coherence length of about 40–60 nm. A scanning electron microscopy (SEM) study confirmed the presence of transparent and wrinkled, dense GO nanosheets among flower-like ZnO nanorods, depending on the GO amounts used in preparation. Raman spectroscopy, Fourier transform infrared spectroscopy (FTIR), ultraviolet–visible (UV–Vis) absorption spectroscopy, and photoluminescence (PL) measurements revealed the impact of GO concentration on the optical and luminescence properties of ZnO NRs/GO nanocomposites. The energy band gap of the ZnO nanorods was independent of GO concentration. Photoluminescence spectra of nanocomposites showed a significant decrease in the intensities in the visible light range and red shifted suggesting a charge transfer process. The nanocomposites’ chromaticity coordinates for CIE 1931 color space were estimated to be (0.33, 0.34), close to pure white ones. The obtained results highlight the possibility of using these nanocomposites to achieve good performance and suitability for optoelectronic applications.

## 1. Introduction

Recent developments in material science and nanostructures have required the synthesis of new versatile materials. With a wide direct band gap (~3.3 eV) and high exciton binding energy (~60 meV) at room temperature, a low cost, and an easy synthesis of different related nanostructures, zinc oxide (ZnO) has emerged as one of the most attractive metal oxides widely used for optoelectronic applications [1,2]. Among ZnO-based nanostructures, the nanorod (NR)-like shape has attracted great attention, due to their properties as well as the low reaction temperature, economic advantages, and facile synthesis [3]. Furthermore, one-dimensional ZnO nanostructures display unique electron transport properties that make them suitable for integration into devices, such as field-effect transistors [4]. The synthesis of nanocomposites based on ZnO and graphene or graphene oxide (GO) appears to be one of the most promising and cost-effective approaches, to control the morphology, band gap, and surface defect states of ZnO nanostructures [5]. Graphene derivative-based structures are key building blocks for many novel technological applications, such as photocatalysts [6], biosensors [7], batteries [8], or supercapacitors [9]. In particular, graphene oxide (GO) has gained attention as a material platform in modern material technology due to its mechanical, electric, thermal, and optical properties that can be readily tailored by a simple chemical functionalization [10,11]. Otherwise, GO is known for its high solubility due to the different functional groups including hydroxyl and epoxide on its surface and also for the inexpensive techniques of production [12], which have facilitated its use in device fabrication [13].

In recent years, many works have been reported about the combination of ZnO nanorods (NRs) with graphene oxide for developing various photonic and optoelectronic applications. Vessali et al. [14] improved ZnO NR/GO layers through a chemical bath deposition (CBD) method, for volatile organic compound detection. Chung et al. [15] developed polysulfone (PSF)-nanohybrid (membranes using a ZnO–GO composite in order to obtain enhanced performance with an improved permeability rate. Rokhsat et al. [16] improved the photocatalytic activity of GO/ZnO nanorod films by UV irradiation. Alamdari et al. [17] presented a cost-effective method for the preparation of a GO–ZnO nanocomposite for a UV detection application. Qi el al. [18] studied the performance of ZnO/GO hybrids as an anode for lithium ion batteries. Khorramshahi et al. [19] reported the synthesis of a Mg-doped ZnO:GO nanocomposite and its properties for acetic acid-sensing applications.

In this study, we prepared ZnO NRs/GO nanocomposites with different GO amounts by a simple hydrothermal process. Different techniques, including X-ray diffraction (XRD), scanning electron microscopy (SEM), as well as Raman, Fourier transform infrared (FTIR), ultraviolet–visible–near-infrared (UV–Vis–NIR), and photoluminescence (PL) spectroscopies, were used to study the effects of GO concentration on surface defects and on the structural, surface morphological, optical, and luminescence properties of ZnO NRs/GO nanocomposites. Our findings contribute to understanding the optical properties of nanocomposites, which is important for the development of promising UV–Vis optoelectronic devices, based on graphene derivatives.

## 2. Materials and Methods

### 2.1. Synthesis of ZnO NR/GO Nanocomposites

**Figure 1:**
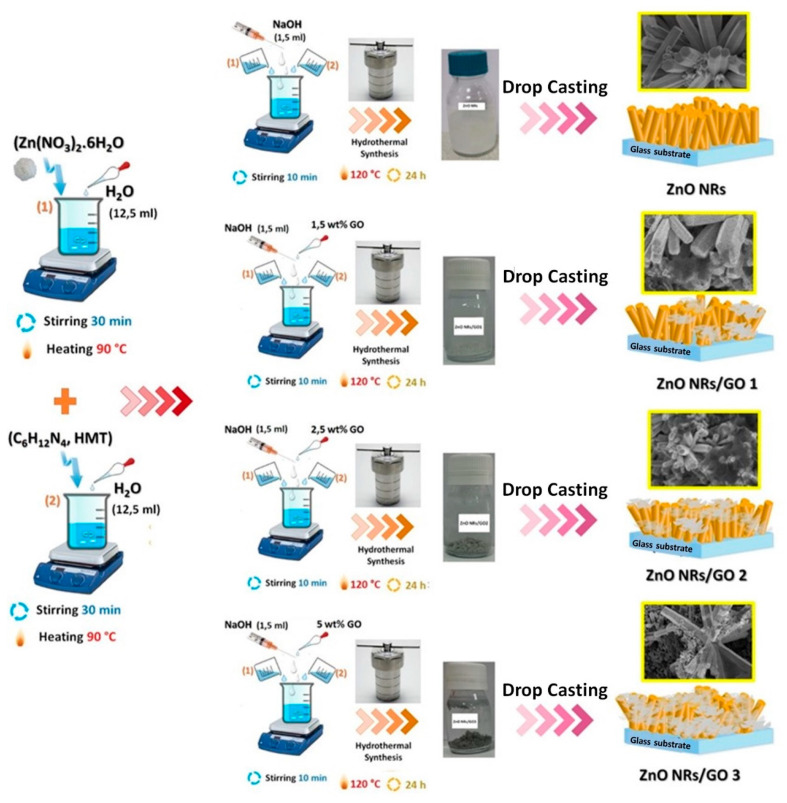
Preparation process of zinc oxide nanorods (ZnO NRs)/graphene oxide (GO) nanocomposites.

ZnO nanorods/GO nanocomposites were obtained using a simple hydrothermal process. The chemicals used in this study were purchased from Sigma-Aldrich Chemie GmbH, Taufkirchen, Germany. First, GO was synthesized using the Hummers method [20]. Second, an equimolar aqueous solution of 0.1 M (Zn(NO_3_)_2_·6H_2_O) and 0.1 M hexamethylenetetramine was dissolved in deionized water. Then, this solution was transferred into a Teflon-lined stainless steel autoclave and heated at 120 °C for 24 h. The ZnO nanorods/GO nanocomposites were synthesized by the same procedure as pure ZnO. The GO solutions with 1.5 wt.%, 2.5 wt.%, and 5 wt.% concentrations were added to this mixture. Finally, the solutions were transferred into a Teflon-lined stainless steel autoclave. Then, the autoclave was kept at 90 °C for 6 h. The as-obtained products were filtered and washed several time using deionized water, and then were casted on glass substrates, and dried at 90 °C for 2 h in air. The synthesis process is presented in Figure 1.

### 2.2. Characterization

The crystal structure of the composites was determined with an X-ray diffractometer (Rigaku Smart Lab system) using CuKα radiation (λ = 1.54178 Å). A field-emission scanning electron microscope (FESEM) JEOL Model JSM 6390F, (JEOL USA, Inc., Peabody, MA, USA) working in high and low vacuum from 0.5 to 30 kV accelerating voltage, equipped with an LaB6 cathode, InLens, and SE2 detectors, and an energy-dispersive X-ray spectrometer (EDX) (Bruker Nano GmbH, Berlin, Germany) were used to characterize the surface morphology of the samples. Raman and FTIR spectroscopic studies were performed in order to analyze the chemical composition in the nanocomposites employing a BRUKER-RFS27 FT-Raman spectrometer (Bruker Optik GmbH, Bremen, Germany) and a PerkinElmer FTIR spectrometer (PerkinElmer, Inc., MA, USA) respectively. A PerkinElmer LAMBDA 45 UV/Vis/NIR spectrometer (PerkinElmer, Inc., MA, USA) was used for measuring the specular transmission. The steady state PL measurements were done using 325 nm UV excitation at room temperature using an Edinburgh FL 920 photoluminescence spectrometer (Livingston, UK) with double monochromators and a 450 W Xe lamp as the excitation source.

## 3. Results and Discussion

### 3.1. XRD Analysis

**Figure 2:**
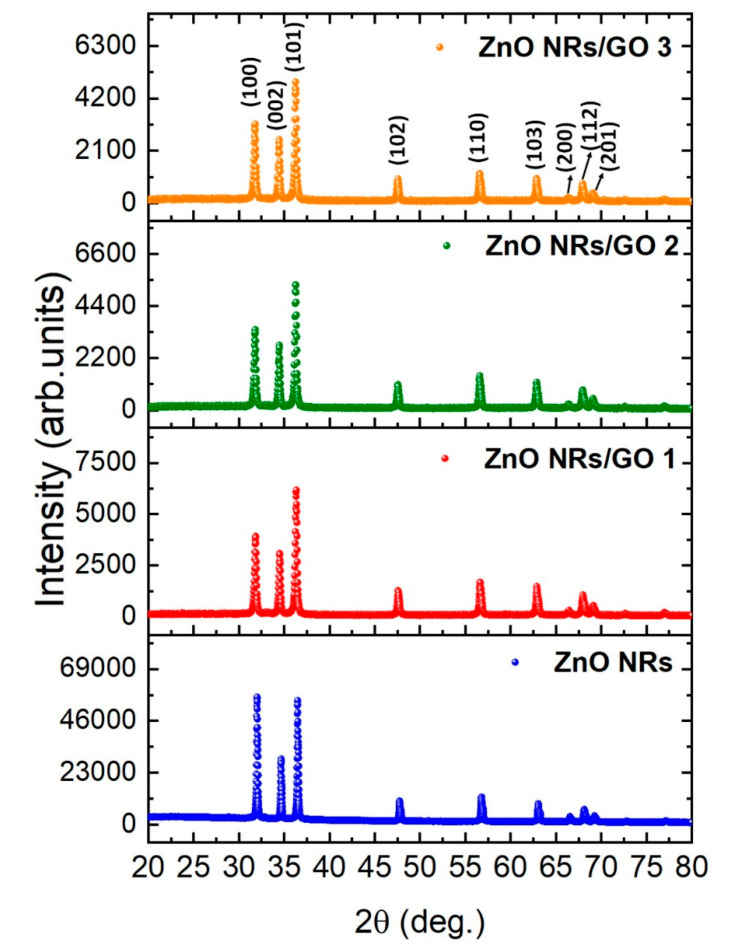
X-ray diffraction (XRD) patterns of ZnO NRs and ZnO NRs/GO nanocomposites with different concentrations of GO.

The XRD analysis was carried out to investigate the crystal structure and phase composition of the ZnO nanorods and ZnO NRs/GO nanocomposites with different concentrations of GO (Figure 2). Both ZnO nanorods and GO/ZnO nanorods exhibit diffraction peaks at 2θ = 31.8°, 34.5°, 36.5°, 47.8°, 56.6°, 62.8°, 66.5°, 67.9°, 69.3°, and 76.9°, assigned to the (100), (002), (101), (102), (110), (103), (200), (112), (201), and (202) crystalline planes, respectively, of hexagonal wurtzite ZnO (JCPDS-ICDD card no. 36-1451), P6_3_mc space group [21,22]. The average crystallite size of ZnO NRs was calculated using the Debye–Scherrer equation of the (101), (002), (101) diffraction planes. The average size of ZnO nanorods within nanocomposites is slightly smaller for increasing GO concentration in comparison with that of ZnO NRs; this might be due to the cleavage of some bigger nanorods during the hydrothermal process [23]. Furthermore, the intensity of the peaks corresponding to ZnO NRs decreased from ZnO NRs to ZnO NRs/GO nanocomposites, which is obviously visible from the XRD pattern and may be due to the functionalization of GO by ZnO nanorods and the decreases in the crystallite size of ZnO.

The variation of lattice parameters of ZnO NRs with different GO concentrations can be calculated by means of a hexagonal lattice relation [24] as follows:

(1) $$\frac{1}{d^{2}}=\frac{4}{3}\left(\frac{h^{2}+hk+k^{2}}{a^{2}}\right)+\frac{l^{2}}{c^{2}}$$

where *d* is the interplanar distance and *h, k*, and *l* denote Miller indices; a and c refer to the lattice constants of the hexagonal structure. The average crystallite size (D), or more precisely the coherent lengths along a crystallographic direction, of ZnO NRs can be calculated using the Debye–Scherrer equation [25] as follows:

(2) $$D=\frac{K\lambda }{\beta \cos\left(\theta \right)}$$

where *k* is a dimensionless shape factor, *λ* is the X-ray wavelength, *β* is the line broadening at half height of the maximum intensity (FWHM), and *θ* is the Bragg angle. The d-spacing values for ZnO nanoparticles were calculated using Bragg’s law (Equation (3)):

(3) nλ=2dsin(θ)

where *n* is a positive integer (equal to 1), *λ* is the wavelength of the incident X-ray beam (*λ* = 1.54178 Å), and *θ* is the angular position of the hkl reflection. The values of lattice constants and crystallite sizes were determined accordingly and are summarized in Table 1.

This 101 interplanar spacing was found to slightly decrease, because of a smaller number of oxygen-containing groups produced by the one-pot hydrothermal treatment during ZnO NRs/GO growth [26,27].

### 3.2. SEM and EDX Studies

**Figure 3:**
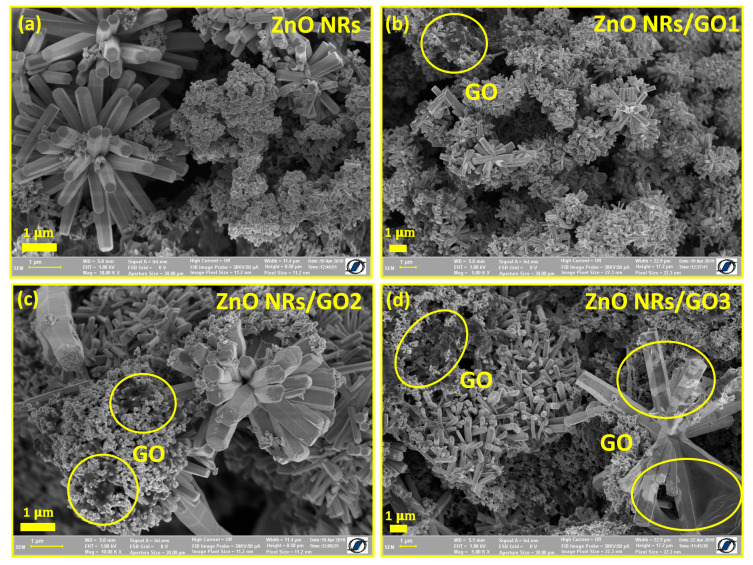
Field-emission scanning electron microscope (FESEM) images of (**a**) ZnO NRs, (**b**) ZnO NRs/GO1, (**c**) ZnO NRs/GO2, and (**d**) ZnO NRs/GO3 nanocomposites.

Figure 3a–d shows high-resolution SEM images of ZnO NRs and ZnO NRs/GO nanocomposites evaluated using JSM 6390 FESEM, working at an applied voltage of 1.8 kV and a working distance of 5 mm. In the absence of GO (Figure 3a), as-obtained ZnO nanorods display a flower-shaped hexagonal section morphology, indicating a high degree of crystallinity. Figure 3b–d shows the presence of several GO nanosheets among flower-shaped ZnO nanostructures. Obviously, GO layers are roughly transparent and, as their amount increases, the layers get thicker because of their tendency to be aggregated by van der Waals forces [28] (Figure 3d). It should also be noted that SEM micrographs show similar morphological features for different ZnO amounts in nanocomposites, depending on the initial GO concentration. By increasing the GO concentration from GO1 = 1.5 wt.% to GO3 = 5 wt.%, the density of the ZnO nanorods decreased. In addition, SEM images of nanocomposites revealed few-layered graphene oxide sheets, no folds with a flat surface, which indicates a high quality of GO. These results indicate the formation of ZnO/GO nanocomposites.

**Figure 4:**
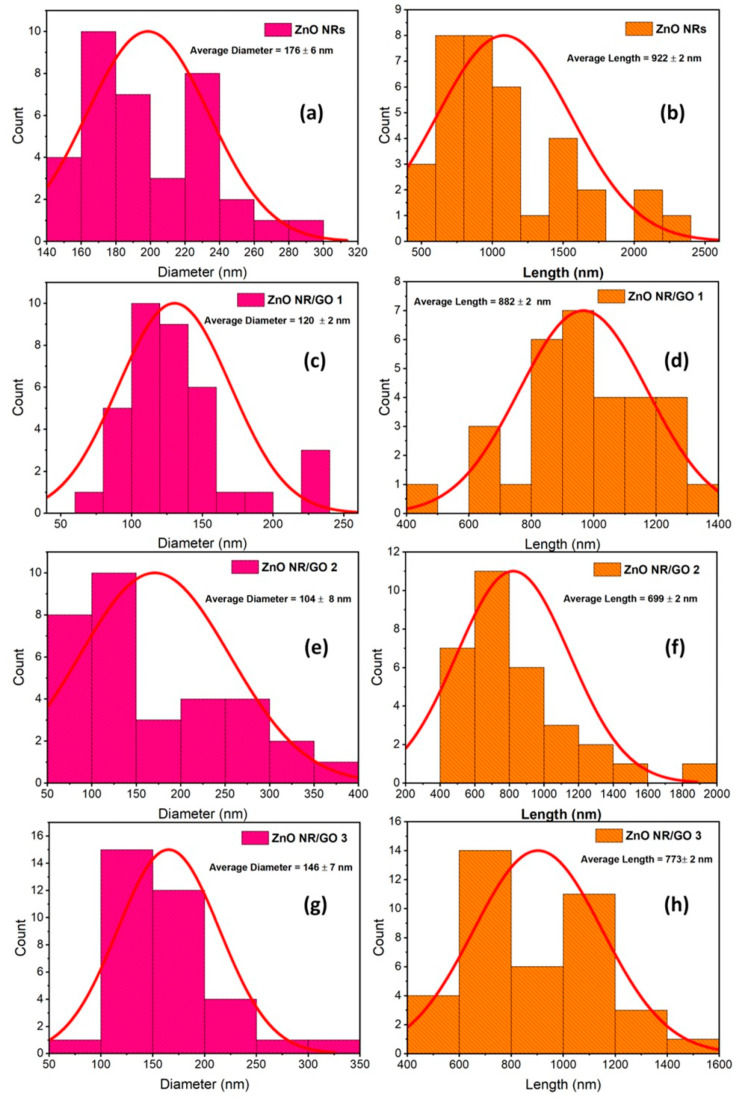
Diameter size distribution (**a**,**c**,**e**,**g**) and length size distribution (**b**,**d**,**f**,**h**) of ZnO NRs, ZnO NRs/GO1, ZnO NRs/GO2, and ZnO NRs/GO3 nanocomposites.

Figure 4 shows the histograms fitted by Gaussian distribution. As can be inferred from this figure, ZnO nanorods display an average diameter of ~175 nm and an average length of ~922 nm (Figure 4a,b). Moreover, the average size in diameter and length of ZnO nanorods in all nanocomposites (Figure 4c–h) is lower compared to those of pure ZnO NRs. Table 1 summarizes the size information for all samples based on SEM characterization and XRD measurements.

**Figure 5:**
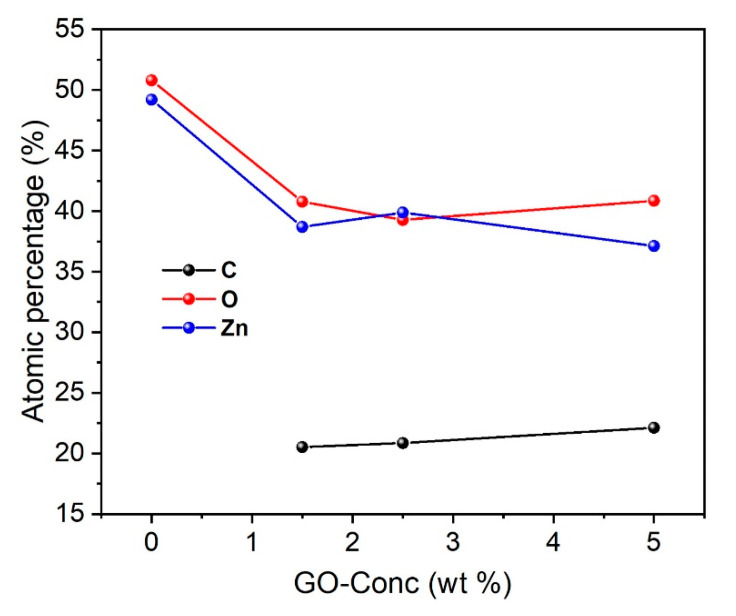
The atomic composition of the ZnO NRs/GO nanocomposites with the different GO concentrations.

The atomic composition of ZnO NRs (Figure 5) is confirmed by EDX analysis. For ZnO NRs/GO nanocomposites, the presence of Zn, O, and C atoms is revealed. The EDX results are in good agreement with the expected chemical composition, which clearly indicates that ZnO/GO nanocomposites were successfully synthesized.

### 3.3. FTIR and Raman Analysis

**Figure 6:**
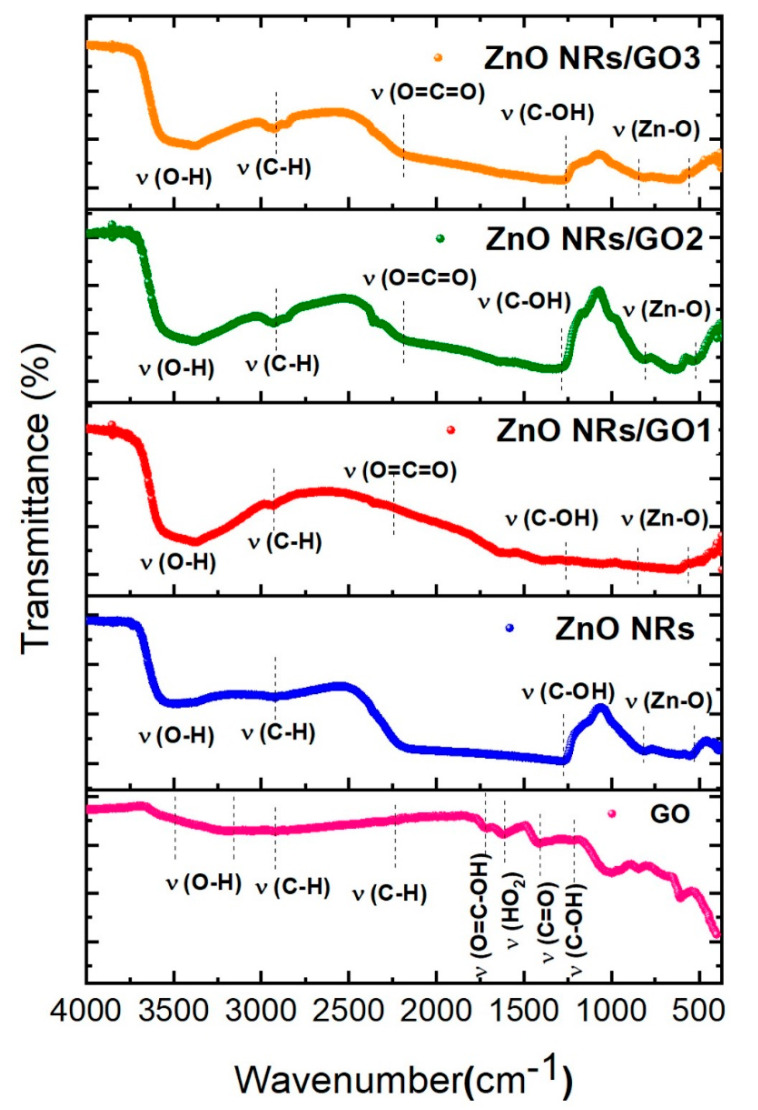
Fourier transform infrared (FTIR) spectra of GO, ZnO NRs, and ZnO NRs/GO nanocomposites with different GO concentrations.

FTIR spectroscopy was used to investigate the surface functional groups in GO layers, ZnO NRs, and ZnO NRs/GO nanocomposites. Figure 6 indicates the presence of eight absorption bands in the FTIR spectrum of GO. The absorption peaks located at 3541–3367 cm^−1^ can be ascribed to O–H group stretching vibration, while the peaks between 2907 and 2232 cm^−1^ correspond to the asymmetric and symmetric stretching vibrations of C–H [28,29]. The bands observed at 1726 cm^−1^, 1615 cm^−1^, and 1416 cm^−1^ are attributed to C=O, C=C, and C–O–C stretching vibrations, respectively [30]. Additionally, the band located at 1222 cm^−1^ can be ascribed to C–OH stretching vibrations [31]. The FTIR spectrum of ZnO NRs comprises several absorption bands (blue curve). The absorption peak at 828–540 cm^−1^ agrees with Zn–O stretching vibration. The absorption peak at 1227 cm^−1^ can be attributed to C–OH stretching vibrations [31]. The peaks around 2905 cm^−1^ are attributable to C–H bonds. The absorption band located at 3421 cm^−1^ corresponds to the hydroxyl group [32]. The FTIR spectra of the ZnO NRs/GO nanocomposites exhibit a number of absorption bands lying in the range from 400 to 4000 cm^−1^. The peaks at 549 and 849 cm^−1^ can be ascribed to Zn–O stretching vibration, thus confirming the presence of ZnO in nanocomposites [33]. Furthermore, the peak observed around 1266 cm^−1^ can be assigned to C–OH stretching vibration [31], that shifted from 1222 cm^−1^ in the case of GO due to composite formation. The absorption peaks at 2949 and 2289 cm^−1^ are attributable to the C–H and O=C=O stretching vibrations, respectively [26], while the absorption band around 3411–3610 cm^−1^ is owing to the O–H stretching vibration. It is recognized that any change in the position and intensity of FTIR peaks of ZnO NRs/GO nanocomposites with respect to those of GO reflects the contribution of functional groups of GO/ZnO nanorods [17].

**Figure 7:**
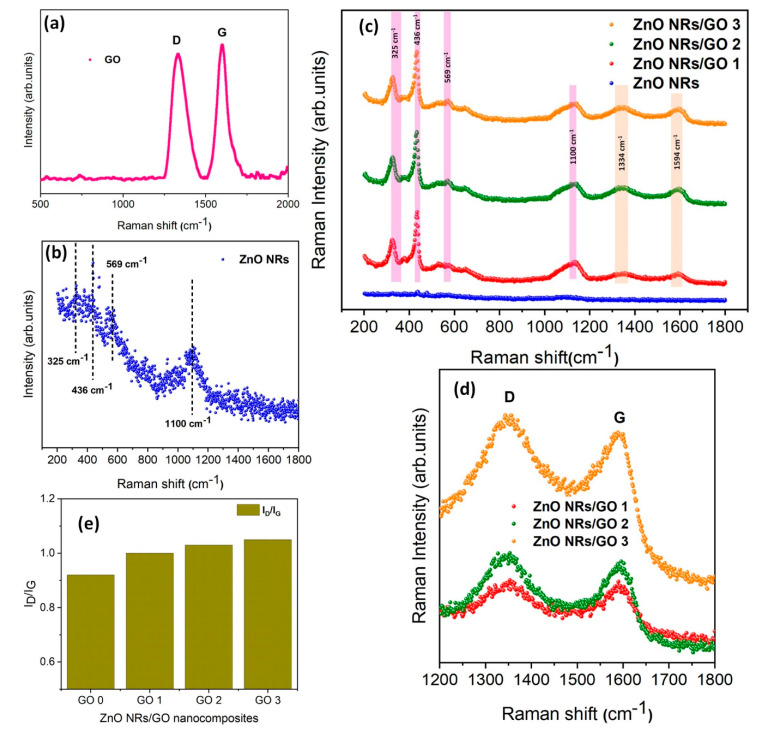
Raman spectra of (**a**) GO, (**b**) ZnO NRs, (**c**) ZnO NRs/GO nanocomposites with different GO concentrations, (**d**) Raman spectra of the same samples shown in the higher region (1200–1800 cm^−1^), and (**e**) the variation of (*I*_D_/*I*_G_) intensity ratio with GO concentrations.

Raman spectroscopy is a powerful characterization technique, particularly useful for further analysis of the structural properties of ZnO and related composites. The GO spectrum (Figure 7a), exhibits two sharp peaks at 1344 cm^−1^ and 1599 cm^−1^, labeled as D and G bands, respectively [34]. The D band is related to a structural disorder of graphene, whereas the G band is assigned to the sp^2^ hybridized carbon atoms (C=C stretching), hence confirming the presence of carbon in nanocomposites [34].

Figure 7b shows the Raman spectrum of ZnO nanorods. The peaks around 325 cm^−1^ and 436 cm^−1^ correspond to the polar and *E*_2_ (high) vibrational modes of the ZnO structure, respectively, confirming the hexagonal wurtzite phase of ZnO nanorods [35]. The peak at ~ 569 cm^−1^ is due to the A_1_ (LO) mode of ZnO [36], whereas the peak found at 1100 cm^−1^ corresponds to the phonon scattering phenomenon [37].

For the ZnO/GO nanocomposites (Figure 7c), all three Raman spectra exhibited identical spectral characteristics, with six leading Raman-active regions, i.e., four regions for the ZnO NRs and the other two regions associated with the GO (D and G bands), which confirms the successful formation of nanocomposites. The only difference was the peaks’ intensity that increased with increasing GO concentration (Figure 7d). Moreover, in the case of the nanocomposite, the D and G bands are shifted to lower wavenumbers, 1334 cm^−1^ and 1594 cm^−1^, compared to GO, due to the hybridization of ZnO.

Furthermore, the peak positions of the D and G bands of GO and ZnO NRs/GO nanocomposites were nearly identical, whereas the intensity ratio of the D and G bands (I_D_/I_G_) changed (Figure 7e): *I*_D_/*I*_G_ = 0.92, 1.00, 1.03, and 1.05 for GO, ZnO NRs/GO1, ZnO NRs/GO2, and ZnO NRs/GO3, respectively. It is well known that the intensity peak ratio of the D and G bands indicates the degree of structural defect concentration in graphitic materials [38]. The increase in the I_D_/I_G_ ratio for ZnO NRs/GO nanocomposites attests that the defect concentration in ZnO NRs/GO is higher than in GO, due to the effect of the hydrothermal treatment on the formation of both ZnO and GO.

### 3.4. UV–Vis Absorption Spectroscopy

**Figure 8:**
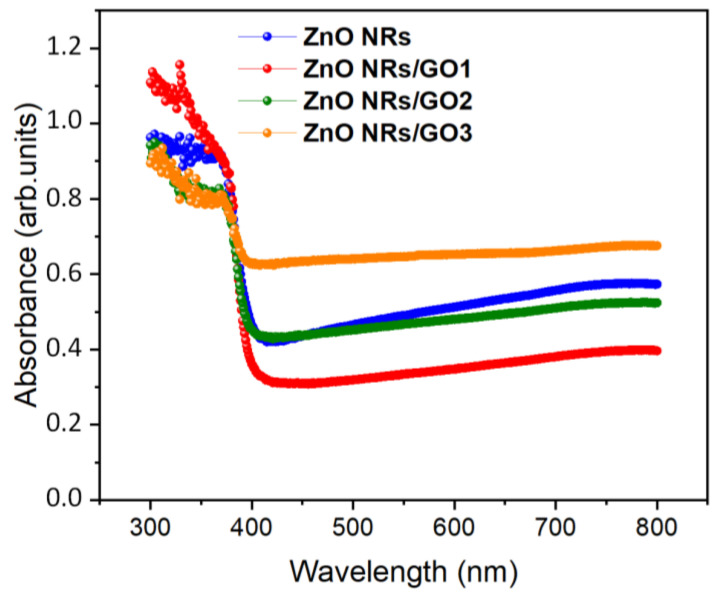
Absorbance spectra of ZnO NRs and ZnO NRs/GO nanocomposites.

The UV–Vis absorption spectra of the ZnO NRs and ZnO NRs/GO nanocomposites with various concentrations of GO are shown in Figure 8. The strong absorption peaks at 366–370 nm, characteristic for all spectra, can be related to the electron transitions from the valence band to the conduction band (O_2p_–Zn_3d_) [38]. No apparent absorption was observed in the visible region. In general, the absorption spectrum of GO exhibits two main particularities, namely the absorption peak at around 230 nm, which is attributed to the π–π* transition, and the absorption peak at around 300 nm, due to the n–π* transition of aromatic C–C bonds [39]. As can be shown, the absorbance of the ZnO NRs/GO1 increases compared to that of the ZnO NRs due to the absorption contribution of GO. Moreover, when compared to ZnO/GO nanocomposites, the absorption edges are gradually redshifted for ZnO NR/GO2 and ZnO NRs/GO3 nanocomposites, due to the interaction of ZnO NRs with GO.

**Figure 9:**
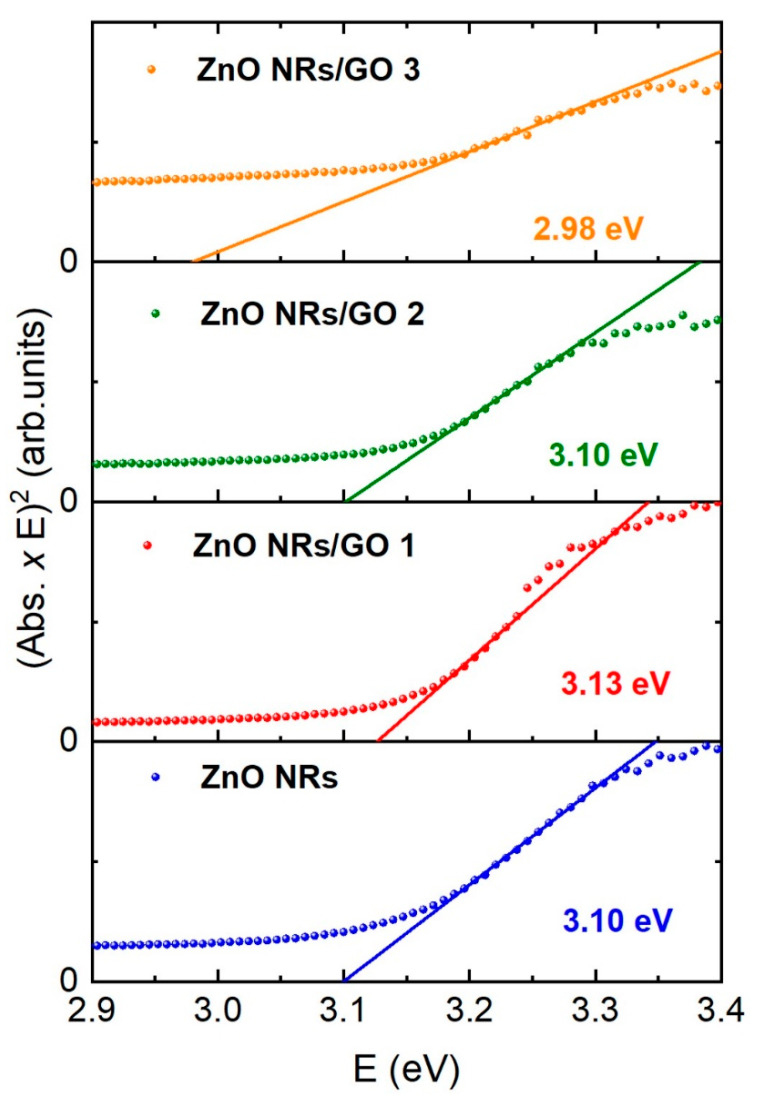
Tauc plots to determine the energy band gaps on the GO amount in ZnO NRs and ZnO NRs/GO nanocomposites.

The optical band gap of the composites was evaluated by a Tauc plot, based on the following relation [40]:

(*αhυ*)^2^ = A (*hυ* − E_g_) (4)

where *hν* is the photon energy, α is the absorption coefficient, A is an energy independent constant, and E_g_ is the band gap. Figure 9 shows the variation of (*αhν*)^2^ versus hν in the fundamental absorption region. The inferred band gap values of the ZnO NRs nanocomposites are around 3.10 eV.

### 3.5. Photoluminescence Spectroscopy

**Figure 10:**
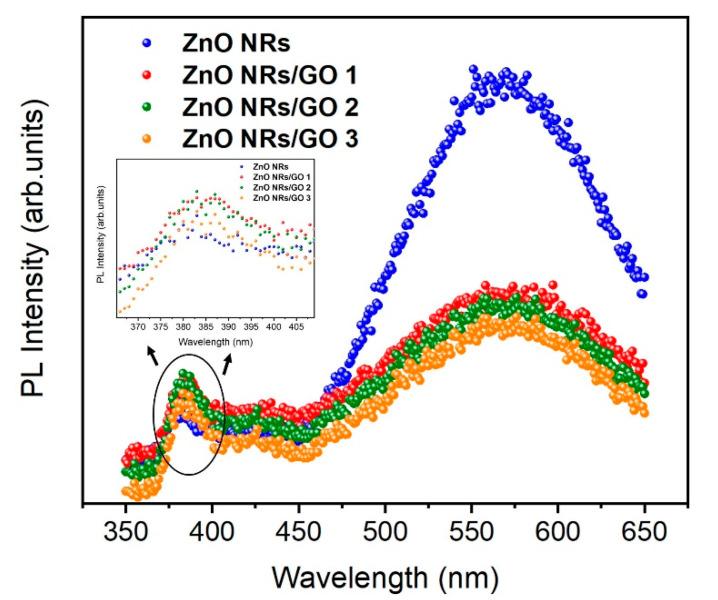
Photoluminescence (PL) spectra of ZnO NRs and ZnO NRs/GO nanocomposites (inset shows room temperature PL spectra in the ultraviolet (UV) region of ZnO NRs and ZnO NRs/GO nanocomposites).

The PL spectra of ZnO NRs and ZnO NRs/GO nanocomposites, recorded with a 325 nm excitation wavelength, are shown in Figure 10. As can be easily ascertained, the samples display three spectrally distinct PL bands—the low intensity band with maximum at 387 nm (UV emission), the low intensity band centered at 425 nm (blue emission), as well as the high intensity and sharp band peaked at 575 nm (green emission). The near-band-edge (NBE) UV emission is due to free ZnO exciton recombination [16]. The blue emission at 425 nm is attributed to the electron transition from a shallow donor level of zinc interstitials (Zn_i_) to the top of the valence band (VB) [41]. The green emission peak observed around 565 nm is due to the transitions from the oxygen vacancy (VO) level of ZnO NRs [42]. Moreover, as shown by PL spectra, the luminescence is substantially quenched after the addition of GO, due to the interaction between excited ZnO NRs and GO. As GO concentration is increased, the PL intensity is decreased, which is caused by the interfacial charge transfer between ZnO NRs and GO layers [43,44].

**Figure 11:**
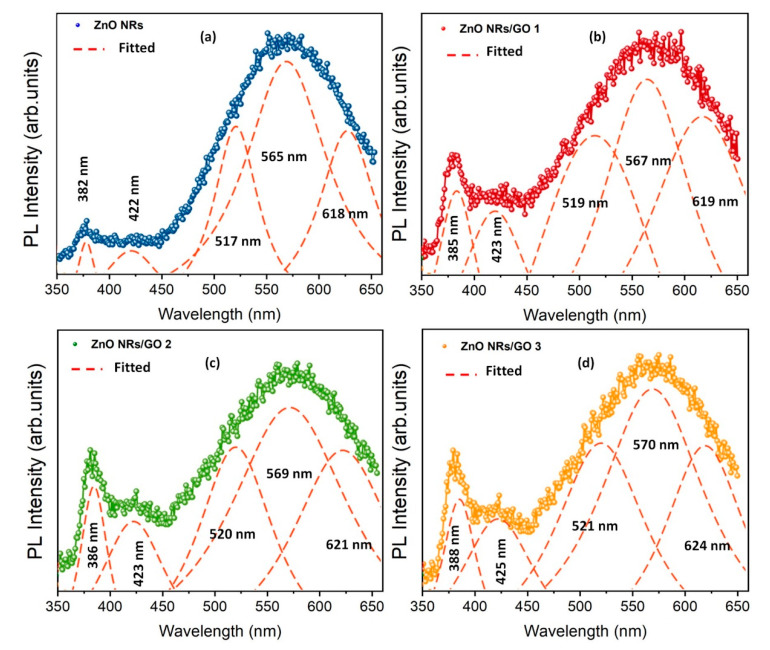
Gaussian line-shape fitting of the PL spectra of (**a**) ZnO NRs, (**b**) ZnO NR/GO1, (**c**) ZnO NR/GO2, and (**d**) ZnO NR/GO3 nanocomposites (solid and dashed lines represent the experimental and the fitted data, respectively).

For a more detailed analysis of emission bands, Figure 11a–d shows the Gaussian fitting of the PL spectra of ZnO NRs and ZnO NRs/GO nanocomposites. In each case, three PL peaks were fitted for the broad visible emission band (in the range of 450–650 nm) and one peak for the UV emission peaked at around 382–388 nm. Apparently, samples exhibit luminescence bands at 517–521 nm that can be related to the emission from defect states due to the presence of oxygen functional groups [45]. Two others peaks at 565–570 nm and 618–624 nm were associated with optical transitions from the VO level and extended oxygen vacancy states to the valance band, respectively [42,46]. It can be also ascertained that when GO’s concentrations increased, emission bands of ZnO NRs/GO exhibited a redshift as compared to those of ZnO NRs. This may be attributed to the interaction between the surface defects of ZnO and the π-electron cloud of the graphene [47]. Studies regarding the theoretical evaluation of optoelectronic properties of 2D materials were published previously. It was demonstrated that localization of electronic states plays a key role in determining the properties of the considered systems [48].

**Figure 12:**
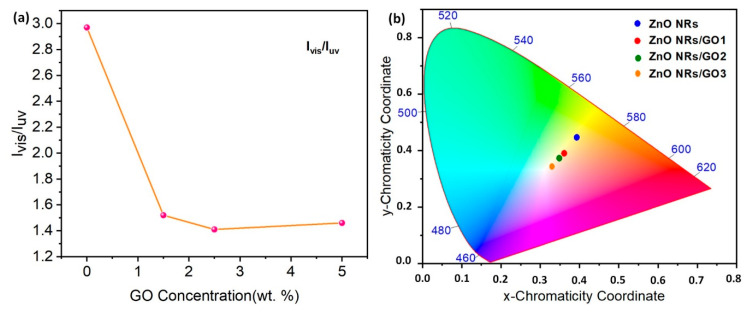
(**a**) The variation of *I*_vis_/*I*_UV_ peak intensity ratio with GO concentration; (**b**) CIE chromaticity diagram for ZnO NRs and ZnO NRs/GO nanocomposites.

The PL intensity of the UV region is increased; however, in the visible region it is decreased by increasing the GO concentration (inset of Figure 10). The *I*_vis_/*I*_UV_ ratio represents one of the important factors for comparing optical characteristics. When comparing the optical properties of nanocomposites, the I_UV_/I_vis_ ratio of ZnO NRs/GO2 means fewer structural defects and a better crystalline quality than the other nanocomposites (ZnO NRs/GO1, ZnO NRs/GO3) (Figure 12a).

However, the emissions have been also characterized by calculating chromaticity color coordinates, using the CIE 1931 chromaticity diagram (Figure 12b). The color coordinates of ZnO NRs were estimated to be about (0.39, 0.44), corresponding to green–orange emission. Clearly, the emission from ZnO nanorods does not belong to the white light region. The obtained color coordinates for ZnO NRs/GO1, ZnO NRs/GO2, and ZnO NRs/GO3 were found to be (0.36, 0.39), (0.35, 0.37), and (0.33, 0.34), respectively, and display a shift towards the white light coordinates (0.33, 0.33) with the increase in GO concentration. We believe that our findings are likely to pave the way for realizing white light emission by means of ZnO/GO nanocomposites. The obtained results could be even used for commercial LED application.

## 4. Conclusions

The nanocomposites were successfully prepared via the hydrothermal process. The XRD measurements indicated the formation of the hexagonal wurtzite structure of ZnO. The SEM and EDX studies revealed the formation of GO layers and flower-shaped ZnO NRs. A decreasing grain size tendency together with increasing amount of GO was observed. The UV-Vis spectra revealed that the nanocomposites exhibited redshifted absorption peaks compared to pure ZnO NRs, with the strongest absorption around 370 nm. The PL spectra of the nanocomposites showed a strong near-band-edge UV emission at 350–400 nm and large visible emission bands at 500–650 nm. The obtained results also indicated the impact of GO concentration on the optical properties of the ZnO NRs. Moreover, the color coordinates of ZnO NRs/GO nanocomposites are close to the white light coordinate (0.33, 0.33).

## Figures and Tables

**Table 1:** Values of average diameter, length, with the standard deviations of the distributions, crystallite size, d-spacing, and lattice parameters of ZnO NRs and ZnO NRs/GO nanocomposites.

		SEM				XRD	
						Lattice	Parameter
Samples	Diameter (nm)	Length (nm)	Shape	Crystallite Size (nm)	d-Spacing (Å)	A = b (Å)	c (Å)
ZnO NRs	~175 ± 3	~922 ± 2	Nanorods/	100-51.34	2.817	3.25	5.20
			nanoflowers	002-60.32	2.604		
				101-45.46	2.478		
ZnO NRs/GO1	~120 ± 2	~882 ± 2	Nanorods/	100-44.93	2.815	3.25	5.20
			nanoflowers	002-60.32	2.603		
				101-45.47	2.476		
ZnO NRs/GO2	~104 ± 8	~699 ± 2	Nanorods/	100-39.94	2.814	3.25	5.20
			nanoflowers	002-45.24	2.601		
				101-40.42	2.476		
ZnO NRs/GO3	~146 ± 7	~773 ± 2	Nanorods/	100-44.93	2.809	3.24	5.20
			nanoflowers	002-40.22	2.598		
				101-40.42	2.472		
